# MESA: automated assessment of synthetic DNA fragments and simulation of DNA synthesis, storage, sequencing and PCR errors

**DOI:** 10.1093/bioinformatics/btaa140

**Published:** 2020-03-04

**Authors:** Michael Schwarz, Marius Welzel, Tolganay Kabdullayeva, Anke Becker, Bernd Freisleben, Dominik Heider

**Affiliations:** b1 Department of Mathematics & Computer Science; b2 Department of Biology, SYNMIKRO, University of Marburg, Marburg D-35032, Germany

## Abstract

**Summary:**

The development of *de novo* DNA synthesis, polymerase chain reaction (PCR), DNA sequencing and molecular cloning gave researchers unprecedented control over DNA and DNA-mediated processes. To reduce the error probabilities of these techniques, DNA composition has to adhere to method-dependent restrictions. To comply with such restrictions, a synthetic DNA fragment is often adjusted manually or by using custom-made scripts. In this article, we present MESA (*Mosla Error Simulator*), a web application for the assessment of DNA fragments based on limitations of DNA synthesis, amplification, cloning, sequencing methods and biological restrictions of host organisms. Furthermore, MESA can be used to simulate errors during synthesis, PCR, storage and sequencing processes.

**Availability and implementation:**

MESA is available at mesa.mosla.de, with the source code available at github.com/umr-ds/mesa_dna_sim.

**Contact:**

dominik.heider@uni-marburg.de

**Supplementary information:**

Supplementary data are available at *Bioinformatics* online.

## 1 Introduction

The ability to synthesize increasingly large artificial DNA constructs, insert these fragments into organisms, and retrieve the genetic information using DNA sequencing led to numerous important discoveries in the biological sciences and opened up new applications for modified DNA, for example, personalized gene therapy (Kitada *et al.*, 2018) and DNA data storage systems (Ceze *et al.*, 2019). The composition of DNA fragments for such applications is still restricted by the limitations of DNA synthesis technologies, cloning methods and the genetic composition of the host organism, while the retrieved information can be corrupted by mutation events or errors during the sequencing process.

Synthetic DNA often has to adhere to a combination of such restrictions, leading to a time consuming and error-prone evaluation process. Depending on the application for the synthetic DNA, it is also often useful to know where and what kind of errors can be expected for a given combination of processes and DNA composition. To ease the evaluation of synthetic DNA and allow user-friendly error simulation, we present MESA (*Mosla Error Simulator*), a web application for the assessment of DNA fragments in terms of guanine-cytosine (GC) content, homopolymer occurrences and length, repeating subsequences and undesirable sequence motifs. Furthermore, MESA contains a mutation simulator, using either the error probabilities of the assessment calculation, literature-based or user-defined error rates and error spectra. MESA is fully customizable using an easy-to-use web interface, without requiring programming experience. All functionality of MESA is also contained in a REST API, enabling the incorporation of MESA evaluations into custom workflows for high-throughput evaluation and error simulation of DNA.

The limitations of the genetic engineering techniques mentioned above can be reduced to limitations regarding GC content, long stretches of a single nucleotide (homopolymers), repeating subsequences and motifs with biological relevance. For example, to synthesize synthetic DNA, *in silico* designed constructs have to be split into smaller fragments [usually 200–3000 base pairs (bp)] (Kosuri and Church, 2014). The fragments are further split into 40–100 bp oligonucleotides (oligos) that are synthesized separately. After synthesis, the oligos are assembled using ligase or polymerase-based methods. Depending on the synthesis method and the overall GC content of a fragment, the GC content of each oligo has to be within a specific range. In oligos with a high GC content, neighboring guanines tend to form an increased amount of hydrogen bonds, leading to inter- and intra-strand folding (Jensen *et al.*, 2010). To assemble oligos into larger fragments, the melting temperature (and thus the GC content) should only deviate slightly between oligos. To adhere to this restriction, the designed DNA fragments should be homogenous with respect to GC content. Homopolymers further increase the synthesis complexity, leading to fragments that are only synthesizable by using modified oligos and more sophisticated assembly methods, resulting in increased synthesis costs.

The amplification of DNA using polymerase chain reaction (PCR) is indispensable for biological science. From DNA synthesis over cloning to DNA sequencing, PCR is used in a wide range of applications. One important factor of a successful PCR is the base composition of the amplification substrate. High melting temperatures due to high GC content of the DNA fragments hinder the separation of strands during the denaturation phase of the PCR. This reduces the yield of the PCR process, since the polymerase cannot efficiently synthesize the growing strand in the presence of previously existing hydrogen bonds. Stretches of repetitive DNA or high GC content can lead to the formation of secondary structures, hindering the elongation of the growing strand. Repetitive regions, as well as homopolymers, can also lead to polymerase slippage, a process in which polymerase briefly loses the connection to the template strand and reconnects at a different position (Fazekas *et al.*, 2010). Further restrictions on the composition of the DNA construct are due to the cloning process: the GC content should be close to the GC percentage of the host genome and motifs used for the cloning process have to be avoided during the design of the DNA construct. The base composition of a DNA fragment is also an important factor for the successful retrieval of genetic information using DNA sequencing technologies. Illumina sequencing, Oxford Nanopore and PacBio sequencing technologies are biased toward DNA with an intermediate GC content, leading to reduced coverage of regions with strongly deviating GC content (Laehnemann *et al.*, 2016). Illumina and Nanopore sequencers also show an increased error rate in the presence of homopolymers (Laehnemann *et al.*, 2016). Depending on the sequencing method used, the resulting data show increased substitution rates for specific DNA patterns: for PacBio data, common substitution patterns are CG → CA and CG → TG, Nanopore data contain an increased amount of TAG → TGG and TAC → TGC substitutions (Weirather *et al.*, 2017) and a common substitution pattern in Illumina data is GGG → GGT (Schirmer *et al.*, 2016).

## 2 MESA usage

**Fig. 1.**
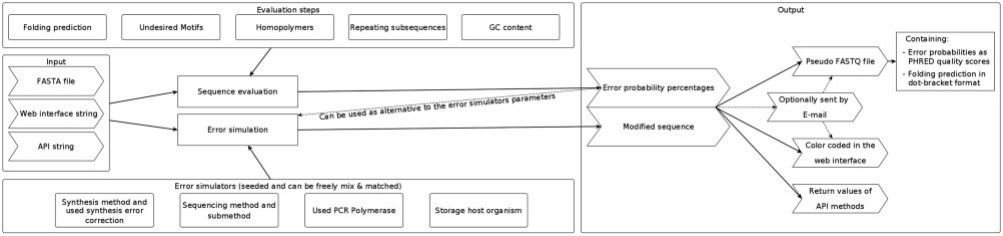
Main workflow of MESA: rectangles denote methods used by the web application and are available in the API, large arrows denote input/output. An extended flowchart of the workflow can be found in the Supplementary Material

The main workflow of MESA is shown in Figure 1. Users can enter any DNA sequence or upload a FASTA file containing multiple sequences. For single short sequences, the results are either shown directly in the web browser or are sent via email when the calculation is finished. If longer and/or multiple sequences are used, notification by email is mandatory. The email contains UUID links for each evaluated sequence as well as FASTQ files of the input and the modified sequence(s) of the error simulation. Calculated error probabilities are displayed in the fourth line of each sequence entry, encoded in ASCII base 33, making them directly comparable to PHRED quality scores. The UUID points to the results page of a single sequence. By default, a UUID link is valid for 365 days, with registered users having the option to change the expiration date or to delete UUIDs of their results. The FASTQ files and UUID links are also available on the results page. Reproducibility is ensured by (i) the ability to download the configuration of the application in JSON format and (ii) by reporting the pseudo-random element of the error simulation in the form of a seed. The configuration file can be uploaded or dragged into the main window, allowing the reuse of prior configurations, while the seed can be entered into the main window to reproduce the results of the error simulation. To save configuration parameters without downloading the configuration file, users can register on the website. Registration is optional and only required for persistent configuration changes, API key generation, a history of completed evaluations and requests to validate user-defined undesired DNA motifs, error rates and error spectra.

A fully documented REST API allows the incorporation of MESA evaluations into custom workflows for high-throughput evaluation and error simulation of DNA.

### 2.1 Error probability estimation

The web interface allows the use of error probability functions for the GC content, length of homopolymers and sequence repeats by manipulation of a graph. The GC error function and the identification of sequence repeats are applied to the complete query sequence as well as to sequence windows of user-defined length. We provide pre-defined error probabilities and descriptions for DNA motifs that are commonly used for cloning or have biological relevance that could hinder the cloning process. The user can adjust the error probability of each motif. It is also possible to add user-defined motifs with custom error probabilities and descriptions. To make a specific motif available for all users of the application, a request for validation can be performed. The validation process involves biologists reviewing each request if (i) the motif has biological relevance and (ii) the motif and the description match. A validation request can be sent via the simulation settings page.

The prediction of secondary structures is based on RNAStructure (Reuter and Mathews, 2010). Users can define the temperature at which the prediction should be carried out. The results are divided into cells for each analysis step and an overall error probability as the sum of all individual error probabilities per DNA region. The regions are color-coded according to their calculated error probability, while hovering over a specific region will show the error probability percentage for it. The predicted secondary structure with the highest probability is shown as dot-bracket notation, in which each base is represented by a character. Dots represent unpaired bases. An open parenthesis represents a base that is paired to another base ahead of it, while a closed parenthesis represents a base that is paired to another base behind it. It is also possible to download the predicted secondary structure as SVG image, PS image, PDF image, CT file or DOT file as well as the PFS distribution file (Supplementary Fig. S2).

### 2.2 Error simulation

MESA also facilitates the simulation of synthesis, sequencing, PCR and storage, and uses either published or user-defined error rates and error spectra. All processes are optional and can be freely mixed and matched, allowing users to customize the simulation to their specific experimental structure and needs.

The available synthesis error rates and spectra are shown in Table 1, containing published error information for different combinations of synthesis methods and error correction methods. The PCR error rates are based on the employed polymerase and the number of PCR cycles that are simulated. We provide error rates for the polymerases *Taq*, *Pfu*, *Pwo* and *Phusion*, which are described by McInerney *et al.*(2014). The storage simulation can be used to simulate mutations of host organisms during an adjustable time interval (Table 2), *in* *vitro* depurination rates calculated using the equation described by An *et al.* (2014) or the Kimura model of molecular evolution (Kimura, 1980). Instead of using published mutation rates, users can also use the binary erasure channel or the additive white Gaussian noise channel for the storage simulation. The DNA sequencing process is simulated using error rates and error spectra of the Illumina single-read and paired-end sequencing methods (Schirmer *et al.*, 2016), PacBio subread and CCS methods (Weirather *et al.*, 2017) and Nanopore 1D and 2D methods (Weirather *et al.*, 2017). The number of errors for a chosen method is calculated by multiplying the sequence length with the raw error rate of the method. The selected type of each error is based on the weights of each error class, positional weights or restrictions and a random number generator. The errors are applied sequentially, allowing for error cross-talk. For example, if a deletion led to the formation of a triplet with a high error probability, this triplet will be included in further error evaluations.

**Table 1.** Pre-defined DNA synthesis error profiles

Method	Error correction
CSO	ErrASE
CSO	MutS
CSO	Consensus shuffle
MBOP	OH
MBOP	HTLH
MBOP	ErrASE
MBOP	NB
MBOP	NGS

*Source:* Data based on Kosuri and Church (2014).

CSO, column synthesized oligos; MBOP, microarray-based oligo pools; OH, oligo hybridization-based error correction; HTLH, high-temperature ligation/hybridization-based error correction; NB, nuclease-based error correction; NGS, NGS-based error correction.

**Table 2.** Pre-defined mutation rates and spectra

Host organism	References
*Escherichia coli*	Lee *et al.*(2012) and Sung *et al.*(2016)
*Saccharomyces cerevisiae*	Drake *et al.*(1998) and Sung *et al.*(2016)
*Mus musculus*	Drake *et al.*(1998) and Sung *et al.*(2016)
*Homo sapiens*	Nachman and Crowell (2000) and Sung *et al.*(2016)

The final sequence including all simulated modifications is shown in a cell of the main results page and can optionally be downloaded in FASTQ format. Modifications are color-coded according to the process in which they occurred, deletions are represented by empty spaces. Hovering over a modified base shows the type of error (insertion, deletion or substitution) and the process that led to it. If multiple modifications of a single base occurred during simulation, the complete sequence of modifications is shown.

For each of the simulated processes, the overall error rate, the distribution of errors between the error types deletions, insertions and substitutions, the rate of errors for each base, substitution patterns and the position of the occurrence of each error type can be adjusted using an intuitive interface. Furthermore, it is possible to send a validation request for custom error rates and error spectra, allowing all users access to the created simulation parameters.

## 3 Customization

The creation of new rules for the error assessment or simulation can be achieved using the rule modification tools of the web application.

### 3.1 Error probability estimation customization

**Fig. 2.**
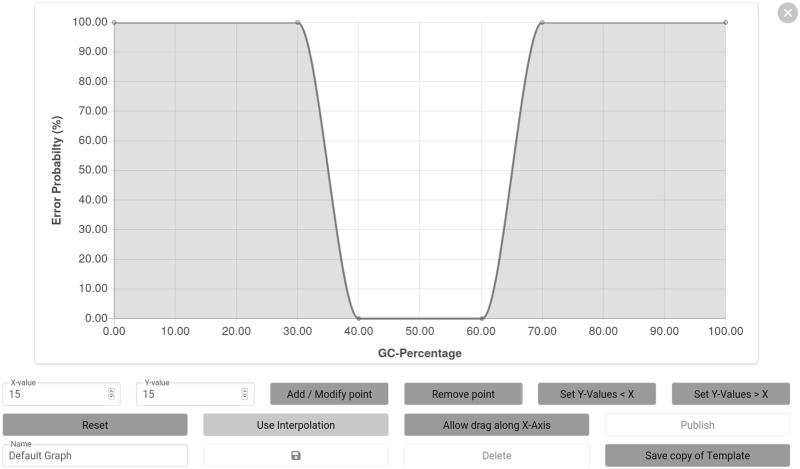
Graph manipulation interface for the creation of custom error probability functions

The web interface supports the creation of error probability functions for the GC content, length of homopolymers and repeating subsequences by either click and drag manipulation of a graph or by using the corresponding input fields (Fig. 2). The vertical positioning (and the horizontal positioning if the ‘Allow drag along *X*-axis’ option is enabled) of each point of the graph can be adjusted. Adding, modifying and deleting points are achieved by specifying the *X*-value of the point and activating the desired function. It is also possible to change the vertical position of multiple points. Custom undesired DNA motifs can be saved in the user profile for subsequent evaluations.

### 3.2 Error simulation customization

**Fig. 3.**
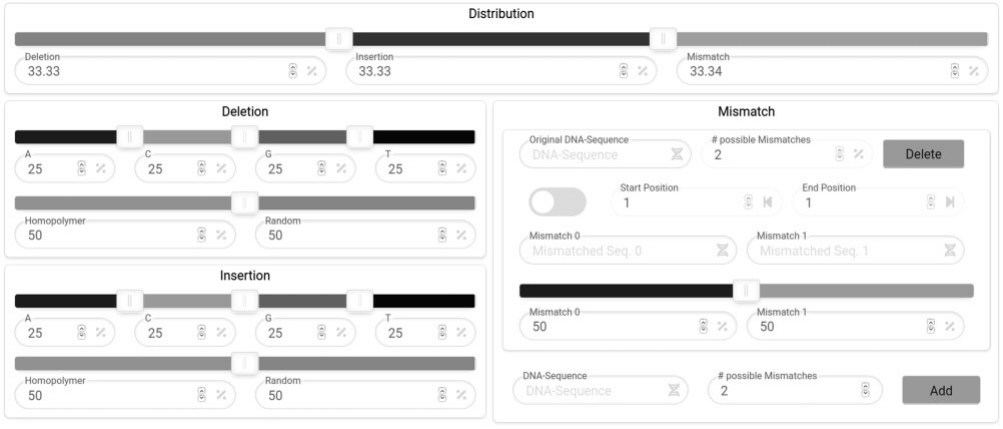
Structure of the simulation rule modification tool for the customization of the DNA error simulation processes

Rules for the simulation of synthesis, PCR, storage or sequencing errors can be added and existing rules can be used as templates for new rules by using the simulation rule modification tool (Fig. 3). A new rule contains at least a name, a raw error rate and the distribution of the different error types (insertion, deletion and substitution). The raw rate in the upper right corner of the tool is the probability of an error event per base. The distribution of the error types is adjusted using the sliders at the top of the tool. For deletions and insertions, sliders on the left side of the tool allow the adjustment of the probabilities to be deleted or inserted for each of the bases, while also allowing to specify the probability that this error occurs in a random position of the input or a homopolymeric region. The right-hand side of the rule modification tool can be used to create substitution rules and positional substitution rules. A substitution rule consists of the target DNA sequence with a length of at least one base, the number of possible substitutions for this target sequence, the sequences for which the target sequence is switched out and the probability for each substitution sequence to be inserted into the input instead of the target sequence. The positional range where substitution occurs can also be defined, which can be used to simulate positional biases in DNA processing methods. An example is given by Schirmer *et al.*(2016), who observed an increased substitution of *T* at position 35 in R2 reads of Illumina data. Depurination rates for *in* *vitro* storage can be calculated for a given pH and temperature by clicking on the *calculate in* *vitro rate* button in the upper right corner of the storage rule customization interface. Equation 1 (An *et al.*, 2014) is used for the calculation of the depurination rates.

(1) $$\begin{array}{c} \text{pH}<2.5,\mathrm{lg}k=14.6-0.707\cdot \text{pH}-\frac{5.63\times 10^{3}}{T}\\ \text{pH}\geq 2.5,\mathrm{lg}k=16.5-0.982\cdot \text{pH}-\frac{5.85\times 10^{3}}{T}, \end{array}$$

where *T* = absolute Temperature (in Kelvin) and *k* = depurination rate per base per second.

## 4 Comparison with other tools

To the best of our knowledge, tools for the evaluation of synthetic DNA are only available from sequencing or synthesis companies. These tools are closed source and specifically designed to evaluate DNA sequences according to the methods used by the company without allowing users to customize evaluation parameters.

Existing tools for the simulation of DNA mutation events in the literature are predominantly used to test the main algorithm of a publication *in silico*. Since these tools are not the main focus, they contain only rudimentary functionality and do not offer customization options. Balado (2013) used the Kimura model of molecular evolution to obtain the Shannon capacity of DNA data embedding. To evaluate two algorithms for data embedding, Haughton and Balado (2013) also used the Kimura model. Heider and Barnekow (2007, 2008, 2011) and Heider *et al.* (2009) used a similar approach, combining either prokaryotic or eukaryotic mutation rates with base-pair specific mutation rates. Moreover, Heider *et al.* (2008) evaluated recombination events in sexually reproducing organisms with respect to DNA watermarking. Nevertheless, these models only account for substitutions of single bases, without accounting for indels and do not include position-based mutation simulation or pattern substitutions. Customization of these tools is strongly limited, tools that contain pseudo-random elements have no mechanisms to ensure reproducibility, and no tool exists that allows users to create and share new rules.

## 5 Validation

User-defined error simulation parameters, undesired subsequences as well as error functions for the homopolymer, GC content and repeating subsequence evaluations can be saved in the user’s profile or exported as a JSON file. It is also possible to request validation for custom content. The following aspects influence the validation process: (i) accompanied references, (ii) biological relevance, (iii) mathematical correctness and (iv) description. Validated content is permanently available for all users, making MESA flexible to be used for different fields and applications.

## 6 REST API

Each registered user can generate an API key to directly access methods used for the evaluation of DNA sequences and the simulation of errors during DNA processing methods. Methods that are available in the API and a brief summary of each method are shown in Supplementary Table S7.

## 7 Deployment and administration

The MESA source code is freely available at github.com/umr-ds/dna_sim, containing instructions for application deployment. After deployment, the first user created will have full administration rights and access to an administration interface with options to confirm or deny validation requests and user management settings.

User information is stored in the Docker network internal PostgreSQL database, with each entry containing (i) a user-ID, (ii) the email address of the user, (iii) a hash value of the user’s password and (iv) information related to the user’s access rights. The algorithm used for password hashing is bcrypt. User accounts can be deleted in the user profile. This action removes all information saved for the corresponding user in the database.

## Supplementary Material

btaa140_Supplementary_Data
