# Supplementary material for: MESA: automated assessment of synthetic DNA fragments and simulation of DNA synthesis, storage, sequencing and PCR errors
Source: Bioinformatics. 2020 Mar 4;36(11):3322–6. doi: 10.1093/bioinformatics/btaa140 (PMC7267826; doi:10.1093/bioinformatics/btaa140)
Supplement: btaa140_Supplementary_Data [file btaa140_supplementary_data.pdf]

Bioinformatics
doi.10.1093/bioinformatics/xxxxxx
Advance Access Publication Date: Day Month Year
Manuscript Category

Sequence Analysis

Supplementary Material for "MESA: automated assessment of synthetic DNA fragments and simulation of DNA synthesis, storage, sequencing, and PCR errors"

Michael Schwarz [1,†], Marius Welzel [1,†], Tolganay Kabdullayeva [2], Anke Becker [2], Bernd Freisleben [1] and Dominik Heider [1,*]

[1] Department of Mathematics & Computer Science and SYNMIKRO, University of Marburg, D-35032 Marburg, Germany
[2] Department of Biology and SYNMIKRO, University of Marburg, D-35032 Marburg, Germany.

[*] To whom correspondence should be addressed.
[†] These authors contributed equally to this work.

Associate Editor: XXXXXXX

Received on XXXXX; revised on XXXXX; accepted on XXXXX

Abstract

Summary: The development of *de novo* DNA synthesis, PCR, DNA sequencing, and molecular cloning gave researchers unprecedented control over DNA and DNA-mediated processes. To reduce the error probabilities of these techniques, DNA composition has to adhere to method-dependent restrictions. To adhere to such restrictions, a synthetic DNA fragment is often adjusted manually or by using custom-made scripts. In this paper, we present MESA (*MOSLA Error Simulator*), a web application for the assessment of DNA fragments based on limitations of DNA synthesis, amplification, cloning, sequencing methods, and biological restrictions of host organisms. Furthermore, MESA can be used to simulate errors during synthesis, PCR, storage, and sequencing processes.
Availability: MESA is available at mesa.mosla.de, with the source code available at github.com/umr-ds/mesa_dna_sim.
Contact: dominik.heider@uni-marburg.de

© The Author 2019. Published by Oxford University Press. All rights reserved. For permissions, please e-mail: journals.permissions@oup.com

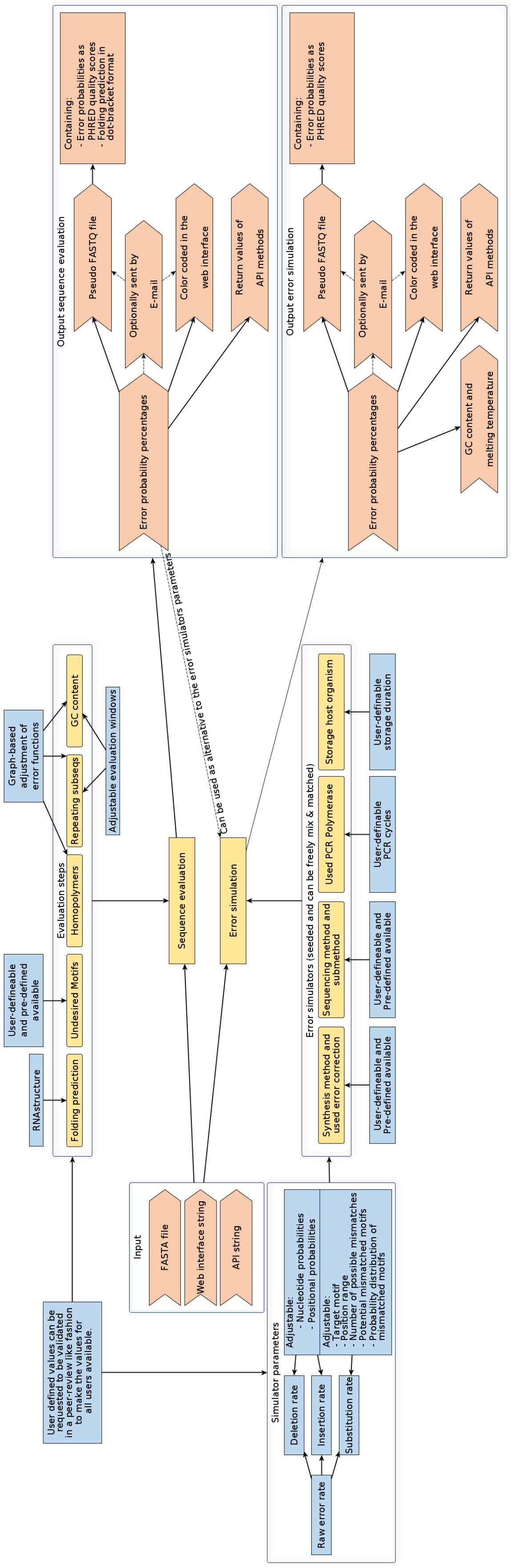

Fig. 1. Workflow of MESA: yellow rectangles denote methods used by the web application and are available in the API, orange arrows denote input / output and blue rectangles denote user-adjustable parameters.

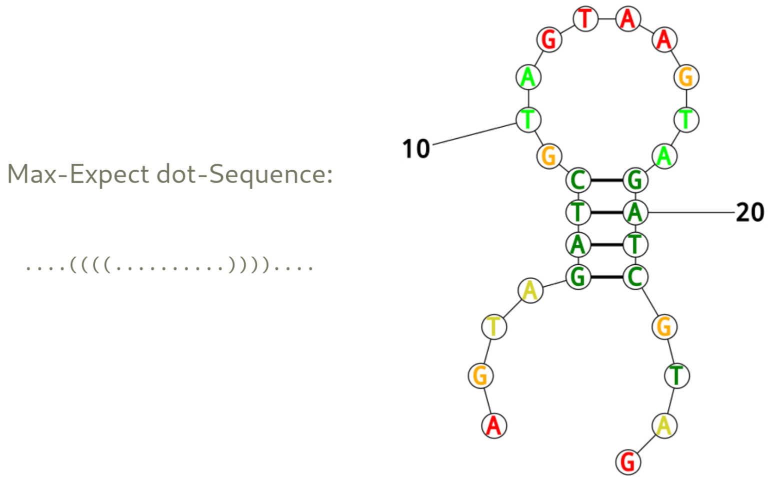

Fig. 2. A predicted secondary structure using the MaxExpect (Reuter and Mathews, 2010) algorithm in dot-bracket format (left) and the same structure as PNG (right).

Table 1. Color codings

Color	Error source	Error type
	Synthesis	Insertion
	Synthesis	Deletion
	Synthesis	Mismatch/Substitution
	Storage	Insertion
	Storage	Deletion
	Storage	Mismatch/Substitution
	Sequencing	Insertion
	Sequencing	Deletion
	Sequencing	Mismatch/Substitution
	PCR	Insertion
	PCR	Deletion
	PCR	Mismatch/Substitution

Table 2. Pre-defined DNA synthesis error profiles

Method	Error-correction	Reference
CSO[1]	ErrASE	Kosuri and Church (2014)
CSO[1]	MutS	Kosuri and Church (2014)
CSO[1]	Consensus Shuffle	Kosuri and Church (2014)
MBOP[2]	OH[3]	Kosuri and Church (2014)
MBOP[2]	HTLH[4]	Kosuri and Church (2014)
MBOP[2]	ErrASE	Kosuri and Church (2014)
MBOP[2]	NB[5]	Kosuri and Church (2014)
MBOP[2]	NGS[6]	Kosuri and Church (2014)

1: Column synthesized oligos, 2: Microarray based oligo pools, 3: Oligo hybridization based error correction, 4: High-temperature ligation / hybridization based error correction, 5: Nuclease based error correction, 6: NGS based error correction

Table 3. Pre-defined DNA sequencing error profiles

Method	Submethods	Reference
Illumina	Single-Read & Paired-End	Schirmer *et al.* (2016)
PacBio	Subread & CCS	Weirather *et al.* (2017)
Nanopore	1D & 2D	Weirather *et al.* (2017)

Table 4. Pre-defined PCR error rates

Polymerase	Reference
Taq	McInerney *et al.* (2014)
Pfu	McInerney *et al.* (2014)
Pwo	McInerney *et al.* (2014)
Phusion	McInerney *et al.* (2014)

Table 5. Pre-defined mutation rates and -spectra

Host organism	Reference
E. coli	Lee *et al.* (2012) and Sung *et al.* (2016)
S. cerevisiae	Drake *et al.* (1998) and Sung *et al.* (2016)
M. musculus	Drake *et al.* (1998) and Sung *et al.* (2016)
H. sapiens	Nachman and Crowell (2000) and Sung *et al.* (2016)

Table 6. Pre-defined in-vitro depurination rates

pH	Temperature (Kelvin)	Reference
8	293.15	An *et al.* (2014)
8	253.15	An *et al.* (2014)
8	193.15	An *et al.* (2014)
7	293.15	An *et al.* (2014)
7	253.15	An *et al.* (2014)
7	193.15	An *et al.* (2014)

Table 7. REST API methods

Endpoint	Description	Input
/api/homopolymer	Calculates homopolymer error probabilities	sequence (string), homopolymer_error_prob (dictionary), asHTML (boolean)
/api/gccontent	Calculates GC error probabilities	sequence (string), gc_windowsize (integer), gc_error_prob (dictionary), asHTML (boolean)
/api/kmer	Calculates error probabilities based on the occurrences of repeating subsequences/kmers	sequence (string), kmer_windowsize (integer), kmer_error_prob (dictionary), asHTML (boolean)
/api/subsequences	Calculates error probabilities based on the occurrences of undesired subsequences	sequence (string), enabledUndesiredSeqs (list of dictionaries), asHTML(boolean)
/api/fasta_all	Wraps do_multiple() which works with FASTA files, calls do_all() for every sequence of the FASTA file in another thread and sends the UUIDs of all results per email	UUID (string), key (string), sequence_list (list)
/api/max_expect	Calculates the secondary structure with the lowest free energy	sequence (string)
/api/getIMG	Loads the image with the given id and type from the Redis server and returns it if possible	ID (string), type (string)
/api/all	Loads the results from the Redis server with the UUID if possible and enables send_mail if the given sequence is longer than 1000 characters.<br>Starts another thread for every request and uses all parameters of the request to calculate the different error probabilities and results	UUID (string), send_mail (boolean), sequence (string), email (string), all parameters oft he request

References

An, R., Jia, Y., Wan, B., Zhang, Y., Dong, P., Li, J., and Liang, X. (2014). Non-enzymatic depurination of nucleic acids: Factors and mechanisms. *PLoS ONE*, **9**(12), e115950.

Drake, J. W., Charlesworth, B., Charlesworth, D., and Crow, J. F. (1998). Rates of spontaneous mutation. *Genetics*, **148**, 1667–1686.

Kosuri, S. and Church, G. M. (2014). Large-scale de novo DNA synthesis: technologies and applications. *Nature Methods*, **11**(5), 499–507.

Lee, H., Popodi, E., Tang, H., and Foster, P. L. (2012). Rate and molecular spectrum of spontaneous mutations in the bacterium escherichia coli as determined by whole-genome sequencing. *Proceedings of the National Academy of Sciences*, **109**(41), E2774–E2783.

McInerney, P., Adams, P., and Hadi, M. Z. (2014). Error rate comparison during polymerase chain reaction by dna polymerase. *Molecular Biology International*, **2014**, 1–8.

Nachman, M. W. and Crowell, S. L. (2000). Estimate of the mutation rate per nucleotide in humans. *Genetics*, **156**, 297–304.

Reuter, J. S. and Mathews, D. H. (2010). RNAstructure: software for RNA secondary structure prediction and analysis. *BMC Bioinformatics*, **11**(1).

Schirmer, M., D'Amore, R., Ijaz, U. Z., Hall, N., and Quince, C. (2016). Illumina error profiles: resolving fine-scale variation in metagenomic sequencing data. *BMC Bioinformatics*, **17**(1).

Sung, W., Ackerman, M. S., Dillon, M. M., Platt, T. G., Fuqua, C., Cooper, V. S., and Lynch, M. (2016). Evolution of the insertion-deletion mutation rate across the tree of life. *G3: Genes, Genomes, Genetics*, **6**(8), 2583–2591.

Weirather, J. L., de Cesare, M., Wang, Y., Piazza, P., Sebastiano, V., Wang, X.-J., Buck, D., and Au, K. F. (2017). Comprehensive comparison of pacific biosciences and oxford nanopore technologies and their applications to transcriptome analysis. *F1000Research*, **6**, 100.
